# A study protocol for a randomised controlled trial evaluating the effects of intraoperative computed tomography on the outcomes of zygomatic fractures

**DOI:** 10.1186/s13063-019-3625-8

**Published:** 2019-08-19

**Authors:** Andrew Higgins, Michael Hurrell, Richard Harris, Geoffrey Findlay, Michael David, Martin Batstone

**Affiliations:** 1 Royal Brisbane and Women’s Hospital, Butterfield Street, Herston, QLD 4029 Australia; 2 Royal Perth Hospital, 197 Wellington Street, Perth, WA 6000 Australia; 3 University of Queensland, St Lucia, QLD 4072 Australia

**Keywords:** Zygomaticomaxillary complex, Zygomatico-orbital, Zygomatic arch, Zygoma, ZMC, ZA, Intra-operative computed tomography, Fracture, O-arm, C-arm

## Abstract

**Background:**

Zygomaticomaxillary complex (ZMC) and zygomatic arch (ZA) fractures are common injuries resulting from facial trauma and frequently require surgical management (Huang et al., Craniomaxillofac Trauma Reconstr 8(4):271-6, 2015). A substantial number of post-operative functional and cosmetic complications can arise from the surgical management of these fractures. These include scarring, inadequate facial profile restoration, facial asymmetries and diplopia (Ellis et al. J Oral Maxillofac Surg 54(4):386-400, 1996; Yang et al. Oral Maxillofac Surg Clin North Am 23(1):31-45, 2011; Kloss et al. Int J Oral Maxillofac Surg 40(1):33-7, 2011). Intuitively, most of these aforementioned complications arise as a result of inadequate fracture reduction; however, current standard practice is to assess reduction post-operatively through plain radiographs or computed tomography (CT) scans. The role of intra-operative CT scanning to assess the reduction of ZMC/ZA fractures and the potential impact on complications, has thus far not been established.

**Methods:**

This is a prospective randomised controlled trial currently being undertaken at the Royal Brisbane and Women’s Hospital. All patients who require operative management of their ZMC or ZA fractures are offered enrollment in the trial. The patients are randomised into two groups: interventional (intra-operative CT) and control (no intra-operative CT). All patients in each group will have post-operative radiographs taken. From these radiographs, the reduction of the ZMC and/or ZA fracture is graded by a blinded assessor. Patients will be reviewed in clinic at 1 week and 6 weeks post-surgery. During these consultations, all patients will be assessed for scarring, diplopia, facial profile restoration and need for revision surgery.

**Discussion:**

Many complications associated with surgical management of ZMC and ZA fractures involve poor aesthetic results as a direct consequence of inadequate fracture reduction. Inadequate fracture reduction is predictable given that small incisions are used and only limited visualisation of the fractures is possible during the procedure. This is due to a desire to limit scarring and reduce the risk of damage to vital structures in an aesthetically sensitive region of the body. It follows that an intraoperative adjunctive tool such as a CT scan, which can assist in visualisation of the fractures and the subsequent reduction, could potentially improve reduction and reduce complications.

**Trial registration:**

Australian New Zealand Clinical Trials Registry, ACTRN12616000693426. Registered on 26 May 2016.

**Electronic supplementary material:**

The online version of this article (10.1186/s13063-019-3625-8) contains supplementary material, which is available to authorized users.

## Background

Zygomaticomaxillary complex (ZMC) and zygomatic arch (ZA) fractures are a relatively common injury in Australia, with the Royal Brisbane and Women’s Hospital managing approximately 160 patients per year [1]. Many of these fractures require surgical reduction and fixation to restore either function or aesthetic form or a combination of the two. Precise reduction of ZA and ZMC fractures can be difficult due to limited visualisation of the fractured bones. Surgical exposure of the fractures is intentionally kept to a minimum to reduce facial scarring and protect vital structures in an aesthetically sensitive region of the body. Current surgical approaches usually produce minimal scarring and provide adequate protection of vital structures; however, as a consequence of this minimalist approach, exposure of the fractures can be very restricted. A potential sequala of minimal fracture exposure is inadequate reduction. This in turn can lead to poor cosmetic results such as facial asymmetry, poor facial profile restoration, facial scarring, limited mouth opening or restricted eye movement [2–4]. It is clearly of the highest priority to surgeons to avoid these potential adverse outcomes.

The current standard procedure for assessing fracture reduction is to perform plain radiography or computed tomography (CT) in the post-operative setting. This provides adequate assessment of the reduction; however, if there are any inaccuracies, correction can only be achieved through a second procedure. With the increasing availability of intraoperative CT, it is surmised that this technology could improve ZMC and ZA fracture reduction and subsequently reduce post-operative complications. To date there are only a small number of studies that have investigated the use of intra-operative CT in the management of ZMC fractures [5–14]. Most of the studies involve small patient sample sizes and none provide level-1 evidence. Many of these studies conclude that intraoperative CT can improve fracture reduction and revision surgery rates. However, to date there have been no randomised controlled trials (RCTs) published to adequately assess this hypothesis.

The aim of this RCT is to assess the use of intra-operative CT in improving radiographic fracture reduction, clinical outcomes and revision surgery rates in the management of ZMC and ZA fractures. Due to the lack of current literature on assessing the potential for use of intra-operative CT, we feel that an RCT will provide an invaluable contribution to the medical literature.

## Methods

### Study objectives

The primary objective of the trial is to determine if intraoperative CT imaging improves clinical outcomes for zygomaticomaxillary complex and zygomatic arch fractures that are managed surgically. This will be assessed through the following variables:

- Post-operative radiographic reduction adequacy
- Need for revision surgery
- Number of intra-operative re-reductions
- Post-operative diplopia
- Facial profile restoration
- Surgical incision and scarring

The secondary objective of the trial is to ascertain whether intraoperative CT imaging has a significant impact on the length of surgery and whether it affects the outcomes of delayed versus early surgical treatment outcomes [15].

### Study design

**Fig. 1:**
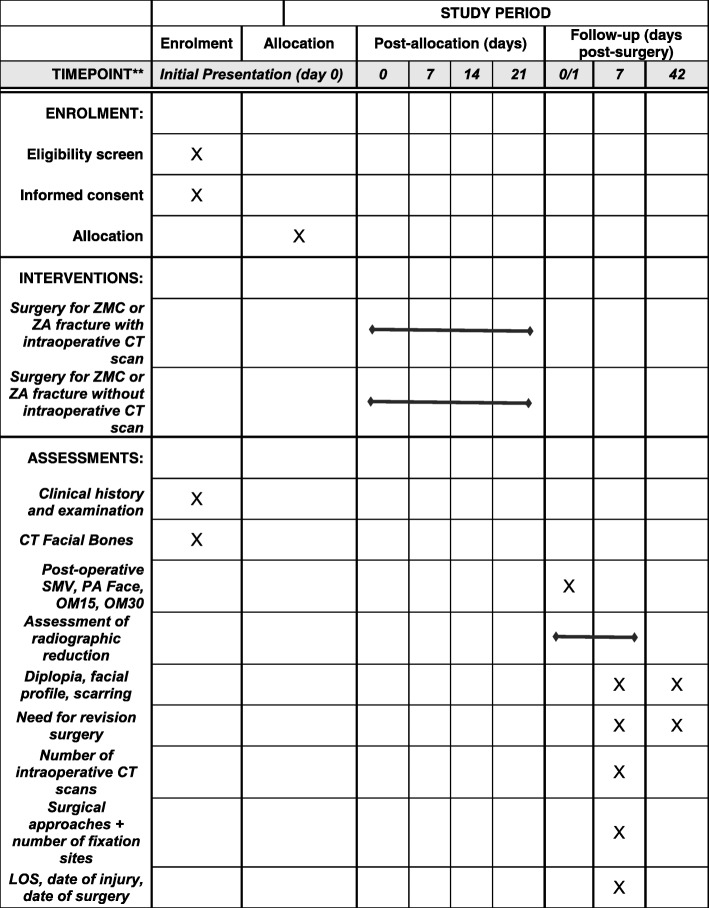
Standard protocol items: recommendation for interventional trials (SPIRIT) figure illustrating the timeline for enrolment, interventions and assessments in the trial. ZMC zygomaticomaxillary complex; ZA zygomatic arch; CT computed tomography; SMV submental vertex; PA posterior anterior; OM15/OM30 occipitomental 15/30 degrees; LOS length of surgery

This is a prospective, single-centre, double-blinded, RCT. It will be conducted by the Oral and Maxillofacial Surgery department (OMFS) at the Royal Brisbane and Women’s Hospital in Brisbane (RBWH), Australia. This is a tertiary teaching hospital and major trauma centre for the city of Brisbane and the state of Queensland. The study was approved by the Royal Brisbane and Women’s Hospital Human Research Ethics Committee (reference number HREC/16/QRBW/18) on 27 July 2016 prior to recruitment of patients. This study complies with the National Health and Medical Research Council’s (NHMRC) National Statement on Ethical Conduct in Human Research (2007). The trial was registered with the Australian and New Zealand Clinical Trials Registry (registration number ACTRN12616000693426) on 26 May 2016 prior to patient recruitment. Please see the Standard protocol items: recommendation for interventional trials (SPIRIT) figure (Fig. 1) for an illustration of the timeline for enrolment, interventions and assessments in the trial. The SPIRIT checklist has been completed for further information (Additional file 1).

If there is any alteration to the trial protocol that has been submitted to the RBWH Human Research Ethics Committee and the Australian and New Zealand Clinical Trials Registry, these organisations will be informed of the amendments immediately.

### Recruitment and consent

All patients who present to the OMFS of the RBWH, with an isolated ZMC fracture or an isolated ZA fracture, will be screened for a position on the trial. As RBWH is an adult hospital, all patients will be 16 years of age or older and they must require surgical management of their ZMC or ZA fracture to be included in the trial. Criteria for exclusion from the trial include the following:

- Pregnancy
- Inability to give informed consent
- Concomitant non-zygomatic facial fractures (including orbital floor fractures) or bilateral zygomatic fractures
- Not wishing to be part of the study

If a patient fulfils the criteria to participate in the trial, they will be informed verbally of what the trial involves, the risks associated with it and their role. They will also be given written information on the trial, the risks involved and all of their rights, including the ability to withdraw at any stage. If the patient is happy to participate, they will be given a consent form to complete. The aforementioned tasks of obtaining informed consent and recruiting patients to the trial will be completed by an oral and maxillofacial surgery registrar (training surgeon) or consultant (surgeon).

### Randomisation

The patients who are recruited to the trial will be randomised into two groups, intraoperative computed tomography scan (intervention) and no intraoperative computed tomography scan (control). The patient will be randomised by the oral and maxillofacial surgery department nurse, who will blindly select a table tennis ball from a container. The container will hold only two balls, with one ball having the word “intervention” and the other ball having the word “control” written on them. Whichever ball is selected by the nurse, is the group to which the patient is allocated.

### Blinding

The patients will not be informed of the group to which they are randomised. Concurrently, the clinician assessing the fracture reduction on the post-operative radiographs will be blinded to the arm of the trial to which the patient has been allocated. The surgeon operating on the patient will not be blinded as this is impossible, given they will be witness to whether or not intra-operative CT is performed. The physician assessing the patient in clinic at both 1 week and 6 weeks post-surgery will not be blinded to the patient’s study group. Blinding of this assessment would be extremely difficult as the physician assessing the patient will be involved with the operation and hence know the allocated group. The clinician assessing the post-operative radiographic reduction will not perform any of the operations in the trial.

### Study groups (control and intervention)

The intervention group will undergo CT during their operation to reduce the ZMC or ZA fracture. This scan occurs after the fracture has been opened, reduced and fixated or in the case of an isolated zygomatic arch fracture, after it has been opened and elevated. Once the scan has been completed the surgeon will review the scan and decide if the reduction is satisfactory. If they are satisfied with the reduction, the incisions will be closed and the operation completed. If there are inaccuracies with the reduction, the fixation equipment will be removed and the fractured bone adjusted to the correct position. It is then left to the discretion of the surgeon to decide if they wish to have another intra-operative CT scan or if they are clinically satisfied with the reduction. Once the surgery is completed, post-operative radiographs will be performed prior to the patient being discharged home. These radiographs will include a submental-vertex view (SMV), a posterior-anterior face (PA face) view and occipitomental 15° and 30° views (OM15 and OM30).

Patients in the control group will undergo their relevant surgery as normal but will not have any intra-operative imaging performed. After completion of the surgery, control group patients will undergo the same post-operative radiography as described for the intervention group. No other concomitant care or interventions are prohibited during the trial.

Surgical incisions are identical in both groups but are at the discretion of the operating surgeon. Direct access to fracture sites is via the upper blepharoplasty incision, subtarsal incision or upper buccal sulcus transoral incision. Elevation is either via a “Gillies” temporal incision or upper buccal sulcus incision.

All procedures will be performed by or under the direct in-theatre supervision of one of three Oral and Maxillofacial Specialist surgeons. All three have extensive experience in the management of facial trauma with each having a minimum experience of managing 300 hundred zygomatic complex fractures.

### Follow up and outcomes

All patients in the trial will have post-operative SMV, PA face, OM15 and OM30 radiographs taken either on the day of surgery or on the following day, prior to discharge. These radiographs will be assessed blinded to study group, by an oral and maxillofacial surgeon who will not be involved in any of the operations or pre-surgical or post-surgical clinical assessments. The adequacy of the fracture reduction will be based on the radiographs and the quality of the reduction will be evaluated as “good”, “fair” or “poor”:

- Good: equivalent to premorbid
- Fair: minor discrepancy but unlikely to require re-operation
- Poor: major discrepancy, re-operation required

**Fig. 2:**
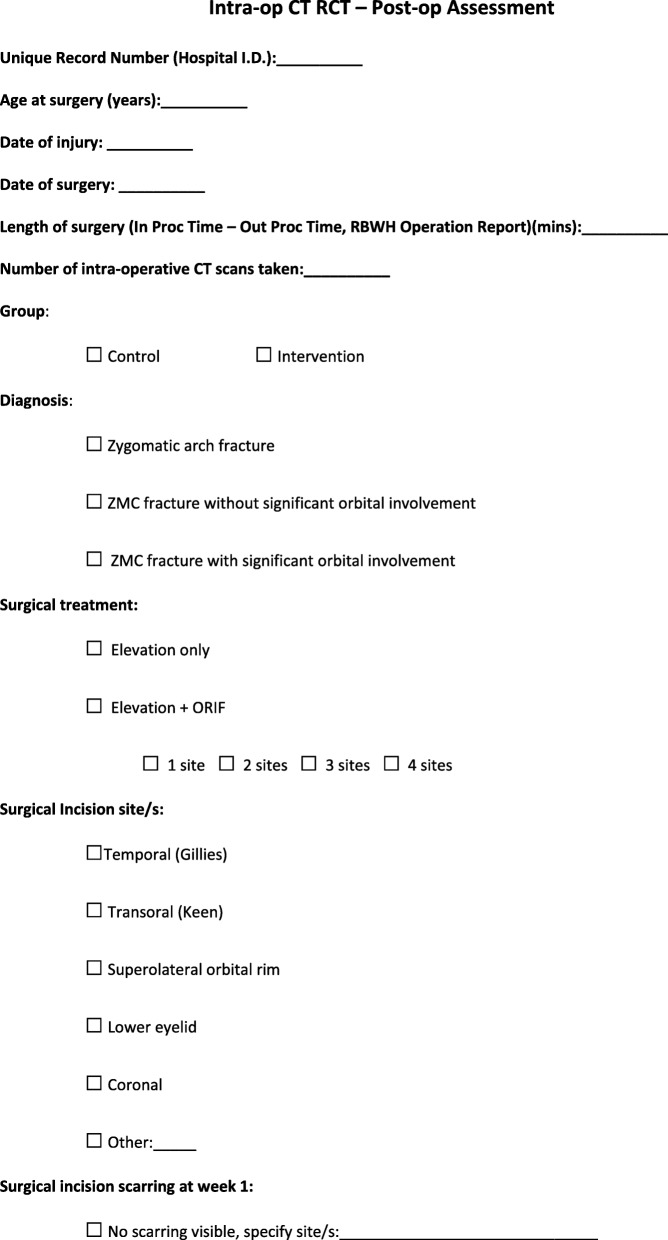
This is the post-operative assessment form that will be completed for all patients. It will be completed when the patient is reviewed in the outpatient department at one and six weeks post-operatively

**Fig. 3:**
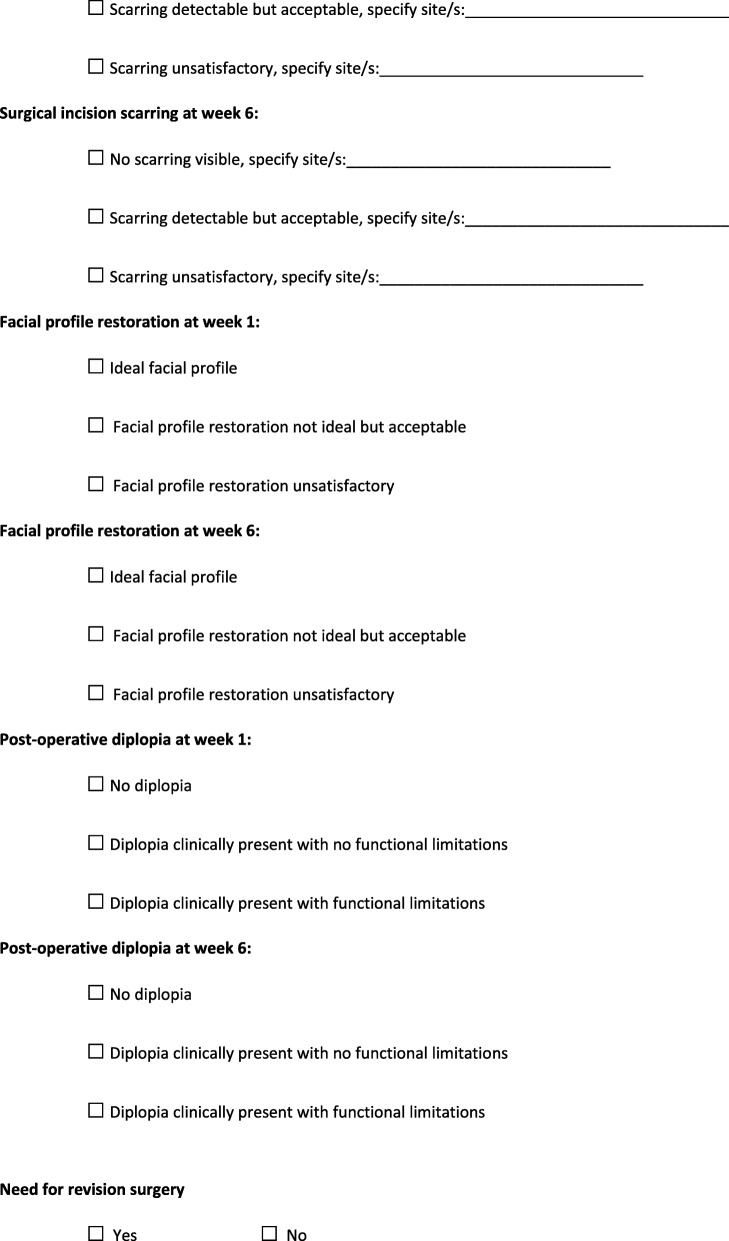
This is the post-operative assessment form that will be completed for all patients. It will be completed when the patient is reviewed in the outpatient department at one and six weeks post-operatively

Patients in the trial who are undergoing ZMC open reduction and internal fixation will stay overnight in hospital, and barring any complications will be discharged home on day 1 post-surgery. Patients undergoing open reduction of their isolated ZA fracture will be discharged on the same day as surgery, unless there are any unforeseen complications. All patients will be followed up in the OMFS outpatient department at 1 week and 6 weeks post-surgery. During these outpatient appointments, the registrar or consultant reviewing the patient will assess them for diplopia, surgical site scarring and facial profile restoration. These parameters will be graded by the OMFS registrar or consultant and their assessment will be recorded on a standardised form (Figs. 2 and 3). The need for revision surgery at any timepoint until 3 months following the completion of the study and a number of other variables (see Figs. 2 and 3 for a full list) will also be recorded on the form.

**Fig. 4:**
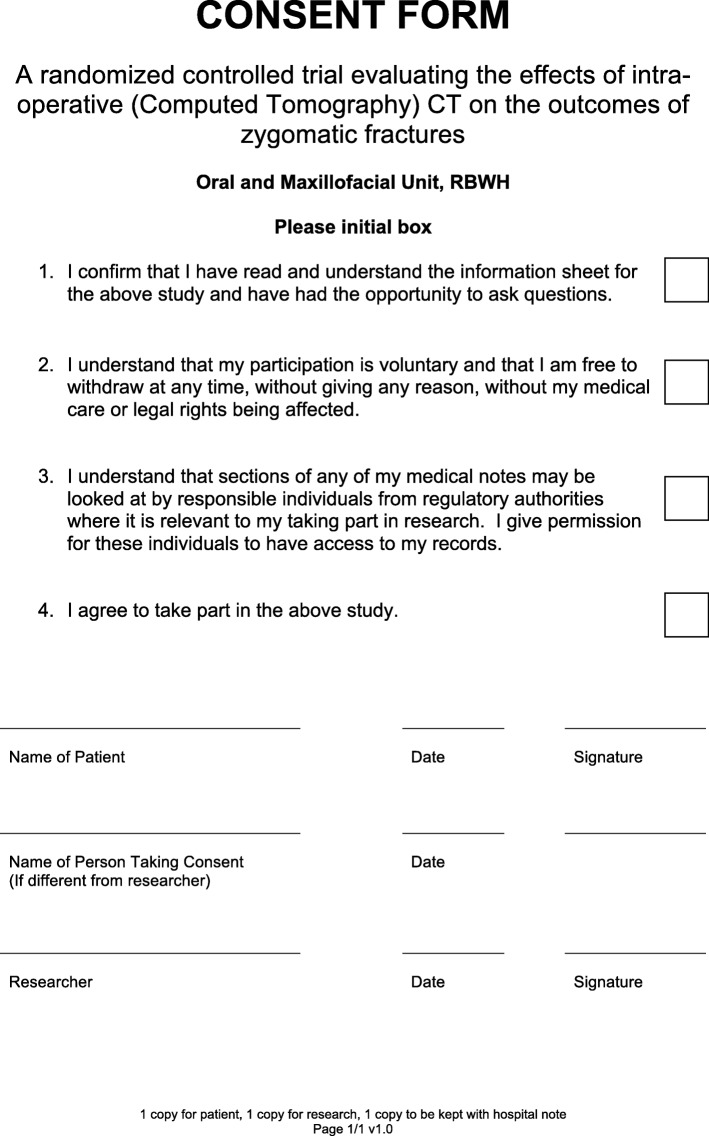
This is the consent form that will be completed and signed by all patients for enrolment into the trial

### Data collection, management and confidentiality

Data from the post-operative radiographic assessments will be entered directly into the trial Excel spreadsheet. All data recorded on the post-operative assessment forms (Figs. 2 and 3) will be transferred to the trial Excel spreadsheet. This Excel file will be stored on a single, password-protected personal computer. At the completion of the trial, the Excel spreadsheet will be transferred to a password-protected computer within the OMFS department at the RBWH. It will be stored on this computer for 15 years. All hard copies of the consent and post-operative assessment forms will be stored in a designated trial folder, which will be stored in a locked room in the OMFS department at the RBWH. At the completion of the trial, this folder will remain in the OMFS department for 15 years. The final trial data set will only be accessible to the authors of the trial.

Data on patients’ names, date of birth, gender, address and occupation will not be collected. Patients will only be identified by their unique record number (URN), otherwise known as a medical record or hospital record number. The URNs will be removed from the data pool prior to statistical analysis.

### Withdrawal

Patients are entitled to withdraw from the trial at any stage. Their withdrawal will have no implications for their ongoing management. Any patient who elects to withdraw will have their trial data deleted and the data will not be used in the statistical analysis.

If trial participants fail to attend their 1-week or 6-week follow-up appointments, any of their previously recorded data will be included in the trial data set and analysed accordingly.

### Evaluation of outcomes

The two-sample *t* test will be used for analysis of continuous variables. The chi-square test will be used for analysis of categorical variables and for all data, a *p* value <0.05 will be considered significant. The software program SPSS will be used for all statistical analysis. Subgroup analysis based on the severity of the fractures will also be conducted. Patients will be analysed as per their treatment but non adherence will be considered in the final presentation of data. An interim analysis will be also be undertaken. There are no other audits of trial conduct planned.

### Adverse outcomes

Any adverse outcomes involving trial participants such as surgical site infection, metal wear infection, excessive bleeding, change in vision, death or common perioperative complications such as deep vein thrombosis, pulmonary embolism or pulmonary infections will be documented and treated accordingly. If warranted, patients will be referred to other medical specialist teams. Any post-trial care and compensation required will be as per Queensland Health standard arrangements.

### Power calculation

An estimated 200 patients will be required for a sample size that will produce significant results. This is based on two-sided testing with a power calculation of 80% and is established from local department outcome estimates and results from studies by Van Hout et al., Hurrell et al. and Van Den Bergh et al. [10, 15, 16]. The variable that required the largest projected sample size to obtain a statistically significant result was the adequacy of post-operative fracture reduction as demonstrated radiographically. The power calculation was performed by Dr Michael David, a biostatistician from the University of Queensland (UQ) School of Population Health.

Due to the low level of evidence available in the current literature, the sample size calculations may not be reliable and estimates were not possible for all variables being proposed in this study. Subsequently, a mid-study analysis will be conducted to more accurately assess the predicted sample size calculations. It is estimated that from the average number patients presenting yearly with ZMC and ZA fractures at the RBWH, this study will take approximately 2 years to complete. If the mid-study analysis identifies a statistically significant result, the study will potentially be terminated early. The decision to terminate the trial or continue after the mid-study analysis will be at the discretion of the study authors.

### Ethical considerations

The standard practice for post-operative assessment following a ZMC or ZA fracture at the RBWH is for a patient to have four plain radiographs (OM15, OM30, PA face and SMV). Occasionally post-operative CT is ordered in lieu of plain radiographs. In this study, the interventional group will undergo intra-operative CT and the control group will not undergo any intra-operative imaging. Both groups will have four plain radiographs taken post-operatively. It is estimated that the interventional group will receive 1.5 millisieverts (mSv) and the control groups will receive 0.4 mSv of ionising radiation. The Australian Radiation Protection and Nuclear Safety Agency (ARPANSA), a department of the Australian Government, estimates that a domestic airline pilot will be exposed to 2 mSv of cosmic radiation per year [17]. ARPANSA also states that there is “no direct evidence of human health effects” up to 10 mSv of ionising radiation [17]. As such, the increased dose of radiation to the interventional group is not considered harmful to the patient and by performing post-operative radiographs on both groups, blinding of the assessing surgeon to group allocation is made possible. If the study shows that intra-operative CT should be used in the management of ZMC/ZA fracture, the need for post-operative imaging would be eliminated, thus reducing the radiation exposure further. This is supported by a paper published by Van Hout el all, which states that “intraoperative imaging rarely increased patient exposure to ionizing radiation as the intraoperative imaging obviates postoperative imaging” [10].

The use of intra-operative CT in the interventional group is likely to increase the overall operative time for these patients. It is estimated that intra-operative CT will increase the operation duration by 10 min.

## Discussion

Surgical management of ZMC and ZA fractures can be complex and challenging due to a number of factors. Visualisation of the fractures is frequently difficult due to the aesthetically sensitive region of the body and critical structures such as the facial nerve. This study aims to determine if better visualisation of the fractures through intra-operative CT will improve fracture reduction and post-operative clinical outcomes. As with all therapies and adjunctive treatments in medicine, the risks and benefits to the patient and cost to the healthcare system must be considered in earnest. Computed tomography does increase the radiation exposure to the patient, albeit by a margin that in considered well below internationally recognised harmful levels. In addition, the act of performing CT will increase operative time. Theatre time is an incredibly valuable and expensive resource with mean costs estimated to be approximately US$37 per minute [18]. Consequently, the implementation of any device that consumes more of this precious resource must be evaluated judiciously and proven to be of benefit prior to any recommendation of regular and widespread use. Conversely, inadequate ZMC and ZA fracture reduction can result in poor cosmetic and functional outcomes for patients [19, 20]. The personal harm to patients of poor fracture reduction and the financial burden placed on hospitals when revision surgery is required cannot be underestimated. As there are no level-1 evidence studies published to answer all of these aforementioned questions, we feel that this RCT is of significant importance in the future management of ZMC and ZA fractures.

## Trial status

The final version of the trial protocol is dated 5 April 2016. Recruitment for the trial began in August 2016 and is estimated to finish in September 2019.

## Additional file

Additional file 1: SPIRIT 2013 Checklist: Recommended items to address in a clinical trial protocol and related documents. (DOCX 41 kb)

## Data Availability

The data collected during the trial will not be made publicly available. At the completion of the trial, the results will be published in full, in a peer-reviewed journal.

## Abbreviations

ARPANSA: Australian Radiation Protection and Nuclear Safety Agency
CT: Computed tomography
mSv: Millisievert
NHMRC: National Health and Medical Research Council
OM15: Occipitomental 15°
OM30: Occipitomental 30°
OMFS: Oral and maxillofacial surgery
PA: Posterior anterior
RBWH: Royal Brisbane and Women’s Hospital
RCT: Randomised controlled trial
SMV: Submental vertex
UQ: University of Queensland
URN: Unique record number
ZA: Zygomatic arch
ZMC: Zygomaticomaxillary complex

## References

[1] Huang W, Lynham A, Wullschleger M (2015). Orbitozygomatic fracture repairs: are antibiotics necessary?. Craniomaxillofac Trauma Reconstr.

[2] Ellis E, Kittidumkerng W (1996). Analysis of treatment for isolated zygomaticomaxillary complex fractures. J Oral Maxillofac Surg.

[3] Yang RS, Salama AR, Caccamese JF (2011). Reoperative midface trauma. Oral Maxillofac Surg Clin North Am.

[4] Kloss FR, Stigler RG, Brandstatter A, Tuli T, Rasse M, Laimer K (2011). Complications related to midfacial fractures: operative versus non-surgical treatment. Int J Oral Maxillofac Surg.

[5] Heiland M, Schulze D, Blake F, Schmelzle R (2005). Intraoperative imaging of zygomaticomaxillary complex fractures using a 3D C-arm system. Int J Oral Maxillofac Surg.

[6] Maheedhar AV, Ravindran C, Azariah ED (2017). Use of C-arm to assess reduction of zygomatic complex fractures: a comparative study. Craniomaxillofac Trauma Reconstr.

[7] Morrison CS, Taylor HO, Collins S, Oyelese A, Sullivan SR (2014). Use of intraoperative computed tomography in complex craniofacial trauma: an example of on-table change in management. Craniomaxillofac Trauma Reconstr.

[8] Stanley RB (1999). Use of intraoperative computed tomography during repair of orbitozygomatic fractures. Arch Facial Plast Surg.

[9] Singh M, Ricci JA, Caterson EJ (2015). Use of intraoperative computed tomography for revisional procedures in patients with complex maxillofacial trauma. Plast Reconstr Surg Glob Open.

[10] Van Hout WM, Van Cann EM, Muradin MS, Frank MH, Koole R (2014). Intraoperative imaging for the repair of zygomaticomaxillary complex fractures: a comprehensive review of the literature. J Craniomaxillofac Surg.

[11] Wilde F, Lorenz K, Ebner AK, Krauss O, Mascha F, Schramm A (2013). Intraoperative imaging with a 3D C-arm system after zygomatico-orbital complex fracture reduction. J Oral Maxillofac Surg.

[12] Shaye DA, Tollefson TT, Strong EB (2015). Use of intraoperative computed tomography for maxillofacial reconstructive surgery. JAMA Facial Plast Surg.

[13] Hoffmann J, Krimmel M, Dammann F, Reinert S (2002). Feasibility of intraoperative diagnosis with a mobile computed tomography scanner. Mund Kiefer Gesichtschir.

[14] Imai T, Michizawa M, Fujita G, Shimizu H, Ota Y, Kitamura T (2011). C-arm-guided reduction of zygomatic fractures revisited. J Trauma.

[15] Hurrell MJ, Borgna SC, David MC, Batstone MD (2016). A multi-outcome analysis of the effects of treatment timing in the management of zygomatic fractures. Int J Oral Maxillofac Surg.

[16] Van den Bergh B, Goey Y, Forouzanfar T (2011). Postoperative radiographs after maxillofacial trauma: sense or nonsense?. Int J Oral Maxillofac Surg.

[17] Australian Radiation Protection and Nuclear Safety Agency. Fact sheet – Ionising radiation and health. 2015. https://www.arpansa.gov.au/sites/default/files/legacy/pubs/factsheets/IonisingRadiationandHealth.pdf. Accessed 17 Jan 2016.

[18] Childers CP, Maggard-Gibbons M (2018). Understanding costs of care in the operating room. JAMA Surg.

[19] Zhang X, Ye L, Li H, Wang Y, Dilxat D, Liu W (2018). Surgical navigation improves reductions accuracy of unilateral complicated zygomaticomaxillary complex fractures: a randomized controlled trial. Sci Rep.

[20] Lee EI, Mohan K, Koshy JC, Hollier LH (2010). Optimizing the surgical management of zygomaticomaxillary complex fractures. Semin Plast Surg.

